# The efficacy of fluoropyrimidine-based adjuvant chemotherapy on biliary tract cancer after R0 resection

**DOI:** 10.1186/s40880-017-0182-y

**Published:** 2017-01-13

**Authors:** Young Saing Kim, Chi-Young Jeong, Haa-Na Song, Tae Hyo Kim, Hong Jun Kim, Young-Joon Lee, Soon Chan Hong

**Affiliations:** 1 Division of Hematology and Oncology, Department of Internal Medicine, Gachon University Gil Medical Center, Incheon, 405-706 South Korea; 2 Department of Surgery, Institute of Health Sciences, Gyeongsang National University Hospital, Gyeongsang National University School of Medicine, 79 Gangnam-ro, Jinju, 660-702 South Korea; 3 Department of Internal Medicine, Institute of Health Sciences, Gyeongsang National University Hospital, Gyeongsang National University School of Medicine, Jinju, 660-702 South Korea

**Keywords:** Biliary tract cancer, Adjuvant chemotherapy, Fluoropyrimidine, R0 resection, Prognosis

## Abstract

**Background:**

The optimal treatment strategy for biliary tract cancer (BTC) after curative-intent resection remains controversial. The purpose of this study was to evaluate the efficacy of fluoropyrimidine-based adjuvant chemotherapy for BTC patients undergoing microscopically margin-negative (R0) resection.

**Methods:**

We retrospectively analyzed the clinical data of BTC patients who underwent curative-intent R0 resection. Patients were eligible if they received either fluoropyrimidine-based adjuvant chemotherapy or observation after R0 resection.

**Results:**

A total of 153 patients were included. In the entire patient cohort, no significant differences were observed in 5-year overall survival (OS) rates (48.4% vs. 39.6%, *P* = 0.439) or 3-year recurrence-free survival (RFS) rates (49.1% vs. 39.5%, *P* = 0.299) between patients who received fluoropyrimidine-based adjuvant chemotherapy or observation. However, for patients with stages II and III BTC, chemotherapy significantly improved 5-year OS rate (52.4% vs. 35.6%, *P* = 0.002) and 3-year RFS rate (55.5% vs. 39.1%, *P* = 0.021) compared with observation.

**Conclusion:**

Fluoropyrimidine-based adjuvant chemotherapy may prolong the survival of patients with stages II and III BTC after R0 resection.

## Background

In western countries, biliary tract cancer (BTC) is a rare disease [1, 2]. However, its incidence is high in South Korea, where it accounts for 2.4% of all diagnosed cancers and is the sixth leading cause of cancer-related death [3]. Patients with BTC usually have a poor prognosis, and only 10%–35% of them have a chance to undergo curative-intent resection [4, 5]. Moreover, the recurrence rate is 30%–50%, even after microscopically margin-negative (R0) resection [6–8].

Given the high recurrence rate of BTC, adjuvant chemotherapy is usually administered in clinical practice. However, its exact role has not been determined due to the lack of randomized, prospective studies. Current information on the efficacy of adjuvant chemotherapy on BTC mostly comes from single-arm or retrospective studies. Several studies compared the efficacy of adjuvant chemotherapy with that of surgery alone; however, these studies included heterogeneous patient populations in terms of tumor location, stage, and margin status, and the conflicting results made it difficult for physicians to know who will benefit from adjuvant chemotherapy [9–15]. Some studies showed that patients with high-risk diseases, such as microscopically margin-positive (R1) resection disease, lymph node-positive disease, and advanced-stage disease, were likely to benefit from adjuvant chemotherapy [14, 15]. A meta-analysis, which included 20 studies with 6712 patients, showed that patients with BTC benefited more from adjuvant chemotherapy or chemoradiotherapy than from radiotherapy alone, and that the greatest benefit from adjuvant therapy was observed in patients who underwent R1 resection and/or had lymph node-positive disease [4]. However, even in this meta-analysis, the efficacy of adjuvant chemotherapy for the patients who underwent R1 resection and/or had lymph node-negative disease remains unclear.

In this study, we aimed to evaluate the efficacy of adjuvant chemotherapy in patients with R0-resected BTC.

## Patients and methods

### Patients

We reviewed the medical records of 193 consecutive patients who underwent surgical resection for BTC between March 1999 and December 2013 at Gyeongsang National University Hospital, Jinju, South Korea. BTC was defined as tumors of the gallbladder and the intrahepatic, perihilar, and distal bile ducts, excluding the ampulla of Vater. Patients were eligible for inclusion if they received either fluoropyrimidine-based adjuvant chemotherapy or observation alone after resection. The patients who met one of the following criteria were excluded: (1) underwent R1 or macroscopically positive margin (R2) resection, (2) died of surgical complications within 3 months after resection, (3) treated with adjuvant radiotherapy or chemoradiotherapy, (4) with another primary cancer at the time of BTC diagnosis, and (5) treated with gemcitabine-based adjuvant chemotherapy. This study was approved by the Institutional Review Board of Gyeongsang National University Hospital.

### Treatment

Curative-intent R0 resection was performed in all patients in the present study, and the subsequent adjuvant chemotherapy plan and schedule were decided according to the clinicians’ discretion. Adjuvant chemotherapy was started within 6–8 weeks after surgery. Patients in the adjuvant chemotherapy group were treated with either single fluoropyrimidine-based chemotherapy—including intravenous 5-fluorouracil (5-FU) and oral agents such as doxifluridine, uracil, and tegafur (UFT); capecitabine; and S-1—or combination chemotherapy consisting of 5-FU and cisplatin. Intravenous chemotherapy consisted of 5-FU (450 mg/m^2^ per day) and leucovorin (20 mg/m^2^ per day) for 5 days every 4 weeks for 6 cycles, or 5-FU (1000 mg/m^2^ on days 1–4) and cisplatin (60 mg/m^2^ on day 1) every 3 weeks for 8 cycles. Oral regimens were as follows: doxifluridine, 800 mg/day in two divided doses for 1 year; capecitabine, 1250 mg/m^2^ twice daily on days 1–14 every 3 weeks for 8 cycles; S-1, 40–60 mg twice daily according to body surface area on days 1–14 every 3 weeks for 8 cycles; or UFT, 300 mg/m^2^ per day in three divided doses for 1 year. Patients in the observation group were followed up after surgery without adjuvant chemotherapy or radiotherapy. Regular assessments were performed in each group using comprehensive physical examinations, tumor marker analysis, and computed tomography to detect recurrence in each group.

### Clinical data collection

By medical chart review, data on the patient characteristics, including demographics, tumor location, histology, TNM stage based on the American Joint Committee on Cancer (AJCC) *Cancer Staging Manual* (7th edition), lymph nodal status, preoperative serum carbohydrate antigen 19-9 (CA 19-9) level, and chemotherapeutic agents, were collected. The cut-off value of serum CA 19-9 level was defined as 37 U/mL (the upper limit of normal range). Survival and recurrence data were also obtained from the medical records. Overall survival (OS) was defined as the time from the date of surgery to the date of death or the last follow-up visit. Recurrence-free survival (RFS) was defined as the time from the date of surgery to the date of first recurrence at any site or death. The follow-up consisted of abdominal computed tomography every 6 months during the first 3 years and yearly thereafter. If signs or symptoms indicated a possible recurrence, investigations were then done to verify whether the patient was recurrence-free. The follow-up cut-off date was January 21, 2014. Recurrences were divided into three patterns: locoregional recurrence, distant metastasis only, and both locoregional and distant recurrence. Locoregional and distant recurrences were defined as recurrent disease within and outside 20 mm of the resection margin or regional lymph node, respectively.

### Statistical analysis

Categorical variables are presented as frequencies and percentages, and continuous variables are expressed as means ± standard deviations. Clinical data were compared using the Chi squared test or Fisher’s exact test for categorical variables and the Mann–Whitney *U* test for continuous variables. OS and RFS were estimated using the Kaplan–Meier method and were compared using the log-rank test between two groups. All significant variables in univariate analysis were included in multivariate analysis. Multivariate analysis using the Cox proportional hazards model with entering selection method was performed to adjust for potential confounding factors. The results are presented as hazard ratios (HRs) and 95% confidence intervals (CIs). A two-tailed *P* value <0.05 was considered statistically significant. Missing data were omitted, and the remaining data were analyzed. SPSS software for Windows, version 21.0 (SPSS Inc., Chicago, IL, USA) was used for all statistical analyses.

## Results

### Patient characteristics

A total of 153 patients were included in the study; of them, 89 (58.2%) received fluoropyrimidine-based adjuvant chemotherapy, and 64 (41.8%) were observed after surgery. Forty patients were excluded for the following reasons: R1 or R2 resection (*n* = 23); early death due to surgical complication (*n* = 7); treatment with adjuvant radiotherapy or chemoradiotherapy (*n* = 4); another primary cancer (*n* = 4); and gemcitabine-based adjuvant chemotherapy (*n* = 2). Characteristics of patients in the adjuvant chemotherapy and observation groups are shown in Table 1. Although there were more men in the observation group, no significant differences were observed in age, tumor location, histologic grade, TNM stage, lymph node status, or preoperative serum level of CA 19-9 between two groups. Most tumors were located in the gallbladder (77/153, 50.3%) and intrahepatic bile duct (51/153, 33.3%). In the adjuvant chemotherapy group, doxifluridine was most commonly used (74/89, 83.1%), followed by 5-FU with leucovorin (6/89, 6.7%), 5-FU with cisplatin (4/89, 4.5%), S-1 (3/89, 3.4%), capecitabine (1/89, 1.1%), and UFT (1/89, 1.1%). Sixty-three of the 153 (41.2%) patients experienced a recurrence. The patterns of recurrence and the proportions of patients who received additional cancer treatment after recurrence were not significantly different between two groups (Table 2).

**Table 1:** Comparison of characteristics between two groups of biliary tract cancer (BTC) patients undergoing microscopically margin-negative (R0) resection

Characteristic	Adjuvant chemotherapy (*n* = 89)	Observation (*n* = 64)	*P* value
*Age (years)* ^a^			0.072
Median (range)	64 (36–83)	67 (47–80)	
*Gender*			0.048
Male	50 (56.2)	46 (71.9)	
Female	39 (43.8)	18 (28.1)	
*Tumor location*			0.480
Gallbladder	43 (48.3)	34 (53.1)	
Intrahepatic bile duct	33 (37.1)	18 (28.1)	
Perihilar bile duct	3 (3.4)	5 (7.8)	
Distal bile duct	10 (11.2)	7 (10.9)	
*Histologic differentiation*			0.225
Well	34 (38.2)	25 (39.1)	
Moderate	31 (34.8)	30 (46.9)	
Poor	17 (19.1)	7 (10.9)	
Unspecified^b^	7 (7.9)	2 (3.1)	
*AJCC stage*			0.152
I	26 (29.2)	29 (45.3)	
II	35 (39.3)	20 (31.2)	
III	18 (20.2)	12 (18.8)	
IVA^c^	10 (11.2)	3 (4.7)	
*Lymph node involvement*			0.426
Yes	23 (25.8)	13 (20.3)	
No	66 (74.2)	51 (79.7)	
*CA 19*-*9 (U/mL) (n* = *126)* ^a, d^			0.328
Median (range)	25.3 (0.1–11,150.0)	17.6 (0.6–9700.0)	

*AJCC* American Joint Committee on Cancer (7th edition), *CA 19*-*9* carbohydrate antigen 19-9

^a^Except these, other values are presented as number of patients with percentage in parentheses

^b^Includes undifferentiated carcinoma (*n* = 3), signet ring cell carcinoma (*n* = 2), carcinosarcoma (*n* = 2), pleomorphic carcinoma (*n* = 1), and large cell neuroendocrine carcinoma (*n* = 1)

^c^All patients had intrahepatic bile duct cancers; 12 patients had TxN1M0 disease and 1 had T4N0M0 disease

^d^Baseline CA 19**-**9 levels were available for 77 patients in the adjuvant chemotherapy group and 49 patients in the observation group

**Table 2:** Comparison of patterns of recurrence and post-recurrence therapies between two groups of BTC patients undergoing R0 resection

Item	Adjuvant chemotherapy (*n* = 41)	Observation (*n* = 22)	*P* value
*Pattern of recurrence*			0.893
Locoregional	9 (22.0)	5 (22.7)	
Distant	21 (51.2)	10 (45.5)	
Both	11 (26.8)	7 (31.8)	
*Post*-*recurrence therapy*			
Patients with any post-recurrence therapy^a^	23 (56.1)	9 (40.9)	0.250
Chemotherapy	18 (43.9)	7 (31.8)	0.350
Surgery	3 (7.3)	2 (9.1)	1.000
Radiotherapy	4 (7.3)	1 (4.5)^b^	0.650
Unknown	1 (2.4)	1 (4.5)	1.000

All values are presented as number of patients with percentage in parentheses

^a^Three patients in the adjuvant chemotherapy group and two in the observation group received surgery and chemotherapy after recurrence

^b^This patient underwent concurrent chemoradiotherapy after recurrence

### Survival analysis

With a median follow-up of 61.2 months (range 1.1–178.2 months), 46 patients in the adjuvant chemotherapy group and 20 patients in the observation group died of disease-related causes. In the entire patient cohort, 5-year OS rates (48.4% vs. 39.6%, *P* = 0.439) and 3-year RFS rates (49.1% vs. 39.5%, *P* = 0.299) did not differ significantly between the two groups.

**Fig. 1:**
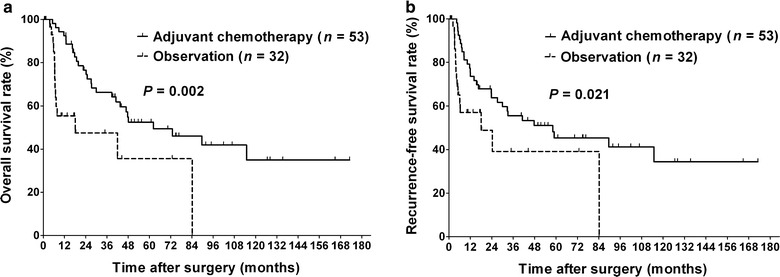
Kaplan–Meier survival curves of patients with stages II and III biliary tract cancer after microscopically margin-negative resection in the fluoropyrimidine-based adjuvant chemotherapy and observation groups. **a** Overall survival. **b** Recurrence-free survival

To assess the relationship between tumor burden and the efficacy of fluoropyrimidine-based adjuvant chemotherapy, we compared the outcomes of patients with various primary TNM stage diseases between the adjuvant chemotherapy and observation groups. In patients with stages I and IV disease, the adjuvant chemotherapy group did not show significant differences in 5-year OS rate compared with the observation group (*P* = 0.612 and *P* = 0.161, respectively); whereas in patients with stages II and III diseases, the adjuvant chemotherapy group had a higher 5-year OS rate than the observation group (52.4% vs. 35.6%, *P* = 0.002; Fig. 1a). The benefit of chemotherapy was similar in terms of RFS. In patients with stages I and IV diseases, no significant difference in 3-year RFS rate was observed between the adjuvant chemotherapy and observation groups (*P* = 0.785 and *P* = 0.116, respectively). However, in patients with stages II and III disease, the adjuvant chemotherapy group showed higher 3-year RFS rate than the observation group (55.5% vs. 39.1%, *P* = 0.021; Fig. 1b).

Additionally, we analyzed the association between the efficacy of adjuvant chemotherapy and other clinical variables (Table 3). The effects of adjuvant chemotherapy appeared to differ depending on tumor locations. In patients with distal bile duct cancer, adjuvant chemotherapy was associated with prolonged OS (*P* = 0.014) and RFS (*P* = 0.016) compared with observation. In patients with gallbladder cancer, adjuvant chemotherapy tended to improve OS rate (*P* = 0.057), but not RFS rate (*P* = 0.148). On the other hand, no significant survival benefit from adjuvant chemotherapy was observed in patients with intrahepatic cholangiocarcinoma or perihilar cholangiocarcinoma. When patients were stratified by other clinical variables such as age, lymph node status, preoperative serum CA 19-9 level, and histology, no significant differences in OS and RFS rates were observed between the adjuvant chemotherapy and observation groups.

**Table 3:** Subgroup analysis of survival between two groups of BTC patients undergoing R0 resection

Variable	5-year OS rate (%)		*P* value	3-year RFS rate (%)		*P* value
	Adjuvant chemotherapy	Observation		Adjuvant chemotherapy	Observation	
*Tumor location*						
Gall bladder	62.5	52.3	0.057	69.7	58.1	0.148
Bile duct	35.2	26.9	0.577	30.8	12.5	0.720
Intrahepatic bile duct	27.5	0	0.265	22.8	32.5	0.549
Distal bile duct	72.9	50.0	0.014	70.0	25.0	0.016
Perihilar bile duct	0	66.7	0.139	0	33.3	0.066
*Age (years)*						
<65	50.3	62.1	0.723	49.4	25.3	0.479
≥65	45.8	25.2	0.570	48.6	41.2	0.508
*Lymph node involvement*						
Yes	23.0	23.8	0.121	21.7	15.6	0.222
No	57.9	39.6	0.587	59.3	44.2	0.336
*CA 19*-*9 level (U/mL)* ^a^						
<37	58.7	75.6	0.195	62.1	76.7	0.430
≥37	34.8	27.9	0.196	32.3	0	0.158
*Histologic differentiation* ^b^						
W/D	71.3	73.5	0.741	68.9	60.3	0.519
M/D or P/D	36.6	29.0	0.476	39.7	24.9	0.325

*OS* overall survival, *RFS* recurrence-free survival, *CA 19*-*9* carbohydrate antigen 19-9, *W/D* well-differentiated, *M/D* moderately differentiated, *P/D* poorly differentiated

^a^Baseline CA 19-9 levels were unavailable for 12 patients in the adjuvant chemotherapy group and 15 patients in the observation group

^b^Seven patients in the adjuvant chemotherapy group and 2 patients in the observation group had unspecified carcinoma

### Multivariate analysis for survival

For 85 patients with stages II and III diseases, lymph node-negative disease, low serum CA 19-9 level, and fluoropyrimidine-based adjuvant chemotherapy were favorable predictors for OS and RFS in univariate analysis. In multivariate analysis, low serum CA 19-9 level and fluoropyrimidine-based adjuvant chemotherapy were independent favorable predictors for OS and RFS (Table 4).

**Table 4:** Multivariate analysis of survival in patients with stages II and III BTC

Prognostic factor	Hazard ratio	95% CI	*P* value
*Overall survival*			
Lymph node (negative vs. positive)	0.461	0.208–1.021	0.056
Serum CA 19-9 level (<37 vs. ≥37 U/mL)	0.379	0.177–0.811	0.012
Treatment (adjuvant chemotherapy vs. observation)	0.231	0.087–0.617	0.003
*Recurrence*-*free survival*			
Lymph node (negative vs. positive)	0.579	0.269–1.250	0.164
Serum CA 19-9 level (<37 vs. ≥37 U/mL)	0.392	0.189–0.811	0.012
Treatment (adjuvant chemotherapy vs. observation)	0.385	0.161–0.923	0.032

*CI* confidence interval, *CA 19*-*9* carbohydrate antigen 19-9

## Discussion

Positive resection margin is considered an important predictor of poor prognosis in BTC patients who undergo surgery [2, 16–18]. Previous studies reported that adjuvant therapy was beneficial to BTC patients with R1 or R2 resection [4, 9, 15, 19]. Takada et al. [9] reported that the 5-year survival rate in patients with stages II–IV gallbladder cancer was significantly higher in the adjuvant chemotherapy group treated with mitomycin C and 5-FU than in the surgery alone group. In the subgroup analysis, the benefit of adjuvant chemotherapy to gallbladder cancer was observed only in patients with non-curative resection, not in those with R0 resection [9]. Another recent study showed the benefits of adjuvant chemotherapy in high-risk BTC patients with high CA 19-9 level, advanced disease, lymph node involvement, and R1 resection, but not in those with R0 resection [15]. Moreover, the two studies did not stratify patients with R0 resection by specific prognostic factors; thus, it was difficult to conclude who would benefit from adjuvant chemotherapy. In our study, we investigated the efficacy of fluoropyrimidine-based adjuvant chemotherapy in BTC patients with R0 resection. When the cohort was analyzed without stratification, no improved survivals were observed in the adjuvant chemotherapy group. However, after stratification, in patients with stages II and III disease, OS and RFS were extended by fluoropyrimidine-based adjuvant chemotherapy compared with observation alone. The efficacy of fluoropyrimidine-based adjuvant chemotherapy in patients with stages II and III BTC who underwent R0 resection was confirmed by multivariate analysis. Similarly, Yamanaka et al. [14] included only patients who underwent R0 resection for BTC and showed that adjuvant gemcitabine chemotherapy for BTC may be effective, particularly for patients with stage III BTC or intrahepatic bile duct cancer. Controversies of adjuvant chemotherapy for R0-resected BTC suggest that patients with other risk factors, such as poor tumor differentiation, lymphovascular invasion, perineural invasion, and lymph node involvement, need to be considered candidates for adjuvant chemotherapy after R0 resection.

Different from previous studies which showed that adjuvant therapy was associated with prolonged survival in patients with lymph node involvement [4, 15, 20, 21], our study showed that lymph node involvement did not identify patients who benefited from fluoropyrimidine-based adjuvant chemotherapy after R0 resection. Lymph node involvement indicates stages IIIB to IV BTC according to the AJCC *Cancer Staging Manual* (7th edition), with the exception of distal bile duct cancer. Since patients with stage IV disease in our study did not benefit from fluoropyrimidine-based adjuvant chemotherapy, lymph node involvement might not be a stratification factor to identify those who will benefit from fluoropyrimidine-based adjuvant chemotherapy. There are several possible reasons why no survival benefit from adjuvant chemotherapy was observed in patients with stage IV disease. First, the number of patients with stage IV disease (*n* = 13) was too small to have a significant difference in survival. Second, the chemotherapies in our study were limited to fluoropyrimidine-based regimens. Given that combination chemotherapy of gemcitabine and cisplatin is considered standard care for patients with advanced BTC [22], fluoropyrimidine-based adjuvant chemotherapy might not be the optimal treatment of patients with stage IV disease, even after R0 resection. Several studies evaluated gemcitabine-based adjuvant chemotherapy. Two Japanese studies showed that, as adjuvant chemotherapy, gemcitabine alone [14] or in combination with S-1 [11] prolonged OS compared with observation in the control group in patients with BTC. Neoptolemos et al. [13] found no difference in survival between adjuvant gemcitabine and fluorouracil plus leucovorin in patients with BTC. However, unlike our study, these studies of gemcitabine-based adjuvant chemotherapy included patients with ampulla of Vater cancer [11, 13, 14], which might have different biologic characteristics compared with BTC [23] or with R1-resected BTC [11, 13]. Comparisons between previous studies and our study are shown in Table 5. Further studies are needed to determine which regimen of adjuvant chemotherapy is more effective for BTC patients with R0 resection.

**Table 5:** Studies of adjuvant chemotherapy for biliary tract cancer

Reference^a^	Year	Design	Resection margin	Tumor location	Stage	Arm	No. of patients	Overall survival		
								Median (months)	5-year rate (%)	*P* value
Takada [9]	2002	Prospective	R0–R2	GB	II–IV	FM Observation	69 43	NA	26.0 14.4	0.0367
Murakami [11]	2009	Retrospective	R0, R1	GB/BD/AoV	II	G + S-1 Observation	50 53	NA	57.0 24.0	<0.001
Neoptolemos [13]	2012	Prospective	R0, R1	BD/AoV	I–IV	FL G Observation	31 34 31	18.3 19.5 27.2	NA	NA
Kobayashi [12]	2012	Retrospective	R0, R1	BD	II–IV	G Observation	51 54	NA	46.0 23.0	0.002
Wirasorn [15]	2013	Retrospective	R0, R1	BD	0–IV	AC Observation	138 125	21.6 13.4	40.1^b^ 29.4^b^	0.01
Yamanaka [14]	2013	Retrospective	R0	GB/BD/AoV	I–III	G Observation	40 158	NA	68.0^b^ 68.7^b^	NS
This present study		Retrospective	R0	GB/BD	II–III	F-based Observation	53 32	NA	52.4 35.6	0.002

*GB* gallbladder, *BD* bile duct, *AoV* ampulla of Vater, *FM* 5-fluorouracil and mitomycin C, *G* gemcitabine, *FL* 5-fluorouracil and leucovorin, *AC* non-specific adjuvant chemotherapy, *F* fluoropyrimidine, *NA* not available, *NS* non-significant

^a^The reference is presented as the first author’s last name followed by the order number of the reference

^b^3-year survival rate

Biliary tract cancer is a heterogeneous disease that is anatomically subdivided. Resectability and surgical management vary depending on the tumor location. Each subtype is considered to have different tumor biology and prognosis [24]. In BTC patients receiving adjuvant therapy, it is also debatable which location of primary tumor is associated with improved survival. Jarnagin et al. [6] suggested that, based on the pattern of initial recurrence, locoregional adjuvant therapy is unlikely to have a significant effect on gallbladder cancer, whereas evidence supports its use in perihilar bile duct cancer. However, other studies showed that adjuvant chemoradiotherapy improved survival in patients with lymph node-positive gallbladder cancer [20, 21] or distal bile duct cancer [25, 26], but not in those with intrahepatic or perihilar bile duct cancer [25]. With respect to chemotherapy, as described above, benefits from adjuvant therapy were seen only in patients with gallbladder cancer [9] or intrahepatic bile duct cancer [14]. By contrast, a meta-analysis showed that there was no difference in survival between patients with gallbladder and bile duct cancers (*P* = 0.68) [4]. In our study, in patients with stages II and III distal bile duct cancer, fluoropyrimidine-based adjuvant chemotherapy was associated with significant improvement in both OS and RFS. However, this result should be interpreted with caution, considering the small number of patients (*n* = 17). Currently, it is difficult to determine which treatment—adjuvant chemotherapy or chemoradiotherapy—is more effective on the primary tumor sites in patients with BTC, especially in those undergoing R0 resection.

The present study had several limitations. First, this analysis was based on retrospective data from a single institution; thus, biases to single institution could not be completely avoided. Second, adjuvant chemotherapy consisted of several different chemotherapeutic regimens. Finally, the small sample size of patients with tumors in each anatomical location was insufficient to generalize our findings. Therefore, a randomized controlled trial comprising a large number of homogeneous patients with BTC is needed to confirm the efficacy of adjuvant treatment.

In conclusion, we found that fluoropyrimidine-based adjuvant chemotherapy improved survival in patients with stages II and III BTC after R0 resection. Patients with stage I disease may not benefit from adjuvant chemotherapy, and other therapeutic strategies may be considered in those with stage IV disease. In addition, the effect of adjuvant therapy appears to differ depending on the primary tumor site. Further large prospective studies are needed to validate our findings and to compare fluoropyrimidine-based adjuvant chemotherapy with other therapeutic options, such as gemcitabine-based chemotherapy and chemoradiotherapy, in BTC patients with R0 resection.
